# Body mass index and risk of connective and soft tissue cancer: results from a large cohort of 1.7 million individuals in Norway

**DOI:** 10.1186/s12885-025-13637-8

**Published:** 2025-02-11

**Authors:** Dagfinn Aune, Marie Nordsletten, Tor Åge Myklebust, Trude Eid Robsahm, Bjørn Steen Skålhegg, Tom Mala, Sheraz Yaqub, Usman Saeed

**Affiliations:** 1 Department of Research, Cancer Registry of Norway, Norwegian Institute of Public Health, Oslo, Norway; 2 Department of Nutrition, Oslo New University College, Oslo, Norway; 3 Department of Epidemiology and Biostatistics, School of Public Health, Imperial College London, White City Campus, 90 Wood Lane, London, W12 0BZ UK; 4 Department of Gastrointestinal and Paediatric Surgery, Oslo University Hospital, Oslo, Norway; 5 Department of Research and Innovation, Møre and Romsdal Hospital Trust, Ålesund, Norway; 6 Division for Molecular Nutrition, Institute of Basic Medical Sciences, University of Oslo, Oslo, Norway; 7 Institute for Clinical Medicine, University of Oslo, Oslo, Norway

**Keywords:** Body mass index, Obesity, Connective and soft tissue cancer, Cohort study

## Abstract

**Background:**

Few studies have investigated the association between adiposity and connective and soft tissue cancer, a rare and understudied cancer entity. We investigated this association in a large cohort of more than 1.7 million individuals in Norway.

**Methods:**

The study cohort included 1,723,692 men and women aged 16–75 years at baseline in 1963–1975. Data on weight and height measurements from the Norwegian Tuberculosis Screening Programme were linked to the Cancer Registry. We estimated hazard ratios (HRs) and 95% confidence intervals (CIs) for the associations between body mass index (BMI) and risk of connective and soft tissue cancer, adjusting for age and sex.

**Results:**

In total 1758 connective and soft tissue cancer cases were registered during 56.1 million person-years of follow-up. The HRs (95% CIs) for the BMI categories of 15-<18.5, 18.5-<25.0 (reference), 25.0-<30.0, 30.0-<35.0, ≥ 35.0 were 0.77 (0.50–1.20), 1.00 (reference), 1.15 (1.03–1.27), 1.28 (1.06–1.54), and 1.69 (1.21–2.37, p_trend_<0.0001). The HR per 5 kg/m^2^ increment in BMI was 1.14 (1.07–1.22) overall, 1.10 (0.99–1.23) in men and 1.17 (1.08–1.27) in women. Obesity in early adulthood (age 16–29 years) showed a suggestive positive association with connective and soft tissue cancer risk with HR (95%CIs) of 1.73 (0.95–3.16), p_trend_=0.09) when compared to normal weight. No clear association was observed between BMI and early-onset (age < 50 years at diagnosis) connective and soft tissue cancer. Positive associations were observed between BMI and soft tissue sarcoma, fibrosarcoma, dermatofibroma, and lipoleiomyoma, but no clear associations were observed with fibrous histiocytoma and liposarcoma.

**Conclusion:**

These results suggest that higher BMI overall and in early adulthood may be associated with increased risk of connective and soft tissue cancer, but BMI seems not to influence early-onset disease. Large cohort studies with more detailed information on confounding factors and BMI trajectories over time are needed for further verification before firm conclusions can be made.

**Supplementary Information:**

The online version contains supplementary material available at 10.1186/s12885-025-13637-8.

## Introduction

Connective and soft tissue cancers, also called soft tissue sarcomas, are rare with incidence rates between 1.8 and 5.6 cases per 100,000 person-years [1–7], and with a male preponderance [2, 3, 6, 7]. Sarcomas span over 80 different histological subtypes [8, 9]. Several studies have suggested increased rates of sarcomas over time [1, 3, 6, 10, 11], pointing to the possible influence of modifiable risk factors. Exposure to radiation, herbicides containing chlorophenol and phenoxyacetic acids, and hereditary syndromes, such as Li-Fraumeni and Werner syndromes are associated with increased risk [11], however, known risk factors account for a small proportion of cases.

The World Cancer Research Fund (WCRF) highlighted in the 2018 Third Expert Report that there is strong evidence linking excess weight to an increased risk of 12 types of cancer, based on findings across 18 evaluated cancers [12]. However, several rare cancers have not yet been evaluated by the WCRF including connective and soft tissue cancer. A large US cohort of 4.5 million veterans found a positive association between a hospital diagnosed obesity and risk of connective and soft tissue cancer in white men with a relative risk (95% confidence interval, CI) of 1.27 (1.09–1.48), but the association was not significant in black men (1.14, 0.72–1.80) [13]. A similar study on hospital diagnosed obesity and cancer risk from Sweden reported a 90% increased risk of connective and soft tissue cancer overall, with a significant increase in women (2.1, 1.1–3.5), but not in men (1.4, 0.4–3.6) [14]. In a Danish cohort study a non-significant positive association (1.19, 0.97–1.44) was reported [15]. Similarly, a positive association was also observed between high body mass index (BMI) and connective and soft tissue cancer in a large Swedish cohort of construction workers, with a hazard ratio (HR) (95% CI) of 1.64 (1.01–2.68) reported for the obese vs. normal weight category [16]. A large pooled analysis of 15 Swedish cohort studies (4.1 million participants, 2029 cases) found a 34% higher risk of connective and soft tissue cancer among persons with obesity when compared to persons with normal weight [17]. An Italian case-control study reported a 3.3–3.5 fold increase in the risk among women and men with obesity, respectively [18], while a US case-control study reported a 2-fold increase in risk with greater body weight [19]. Although previous studies suggests an increased risk with adiposity, several of these studies relied on hospital diagnoses of obesity and did not have information on BMI across the full range [13–15], thus further studies are needed to clarify the potential role of BMI in the development of this disease and any potential dose-response relationships. We utilized a large a large Norwegian cohort study of 1.7 million participants with long-term follow-up to investigate associations between BMI and risk of connective and soft tissue cancer to shed further light on this association. As some studies have suggested a role of BMI in early adulthood in increasing risk of other cancers [20–22] and an association between BMI and early-onset cancers [21, 23, 24], we also investigated the role of early adulthood obesity and age at diagnosis in the association with connective and soft tissue cancer risk.

## Methods

Weight and height measurements from the Norwegian Tuberculosis Screening Program (NTSP) and data on cancer diagnoses from the Cancer Registry of Norway (CRN) were linked. The data from the CRN is complete and of high quality [25]. The NTSP conducted a nationwide survey from 1963 to 1975, wherein healthcare providers measured and registered participants weight and height [26]. We have reported on the association between BMI and several other cancers in this study in several previous publications [21, 27–30].

### Study population

The NTSP included 1,911,598 individuals (aged 7–99 years) who participated in the survey (approximately half the Norwegian population at the time). The current analysis excluded individuals < 16 years and > 75 years age, those with BMI < 15 or > 50 kg/m^2^, and those diagnosed with cancer (except basal cell carcinoma of the skin) at baseline or within the first year of follow-up, individuals with short stature (women < 150 cm and men < 161 cm), those with missing data on weight and height, and those with no follow-up time. Thus 1,723,692 men and women aged 16–75 years at baseline were included in the analysis after all exclusions were made.

### Outcome data

Cancer diagnoses were obtained by linkage to the CRN using the International Classification of Diseases version 10 (ICD-10) codes. Connective and soft tissue cancer cases were identified by ICD-10 code C49. Information on deaths and date of death were obtained from the Cause of Death Registry. Individuals were followed from the NTSP screening date until cancer diagnosis, 75 years of age, death, emigration or the end of follow-up (December 31, 2018).

### Statistical methods

Multivariable Cox proportional hazards regression models were used to estimate HRs (95% CIs) for the association between BMI and connective and soft tissue cancer, adjusting for age groups at the time of screening and sex (for the overall analysis). The underlying time scale was age. The proportional hazards assumption was assessed using Schoenfeld’s test without observing any violations (*p* = 0.61). BMI was categorized according to standard cut-off points 15-<18.5, 18.5-<25, 25-<30, ≥ 30. The latter BMI category was subdivided into 30-<35, and ≥ 35, to assess the impact of more extreme levels of adiposity. Linear trend was tested for by replacing the BMI category with the median value within each category and entering this variable as a continuous variable in the models. We also analysed the association per 5 kg/m^2^ increase in BMI. Sensitivity analyses excluding the first 5 years of follow-up were conducted to consider reverse causation. We conducted separate analyses of the major histological subtypes of connective and soft tissue cancer with at least 100 cases in total. We additionally analysed the association between BMI in young adulthood (ages 16–29 years) and connective and soft tissue cancer as well as between BMI and early-onset (age < 50 years at diagnosis) connective and soft tissue cancer. The statistical analyses were conducted using Stata version 18.1 (StataCorp, College Station, TX) and a two-tailed *p*-value < 0.05 was considered statistically significant.

### Ethical approval

Ethical approval was obtained from the Regional Committee for Medical and Health Research in South-Eastern Norway (REC#: 2018/670), Norwegian Institute of Public Health, Cancer Registry of Norway, Norwegian Tax Administration (which administers the National Population Registry), and Oslo University Hospital data protection officer (SD0759843). This study adhered to the Declaration of Helsinki.

## Results

The cohort included 1,723,692 participants (829,081 men and 894,611 women) aged 16–75 years at baseline. Participant characteristics are shown in Table 1. The mean follow-up was 32.5 (SD 14.8) years and over 56.1 million person-years accrued 1758 incident connective and soft tissue cancer cases.

**Table 1:** Characteristics of the participants in the Norwegian tuberculosis screening program (baseline data included during 1963–1975)

	Men and women	Men	Women
Study cohort	1,723,692	829,081	894,611
Age at baseline (years)	43.2 (16.5)	43.2 (16.5)	43.1 (16.5)
Age group 16–29 years	451,120 (26.1%)	216,228 (26.1%)	234,892 (26.3%)
Height (cm)	168.6 (8.8)	175.2 (6.3)	162.5 (5.8)
Weight (kg)	70.1 (11.9)	74.8 (10.6)	65.6 (11.4)
BMI (kg/m^2^)	24.6 (3.9)	24.4 (3.2)	24.9 (4.4)
BMI categories			
Underweight (15-<18.5)	36,114 (2.1%)	12,297 (1.5%)	23,817 (2.7%)
Normal weight (18.5-<25.0)	988,908 (57.4%)	494,357 (59.6%)	494,551 (55.2%)
Overweight (25-<30.0)	545,538 (31.6%)	282,710 (34.1%)	262,828 (29.4%)
Obese, all (≥ 30.0)	153,132 (8.9%)	39,717 (4.8%)	113,415 (12.7%)
Obese grade 1 (30-<35.0)	124,402 (7.2%)	36556 (4.4%)	87,846 (9.8%)
Obese grade 2 (≥ 35.0)	28,730 (1.7%)	3161 (0.4%)	25,569 (2.9%)

Values are means (SDs) for continuous variables and numbers (percentages) for categorical variables

BMI: body mass index

**Fig. 1:**
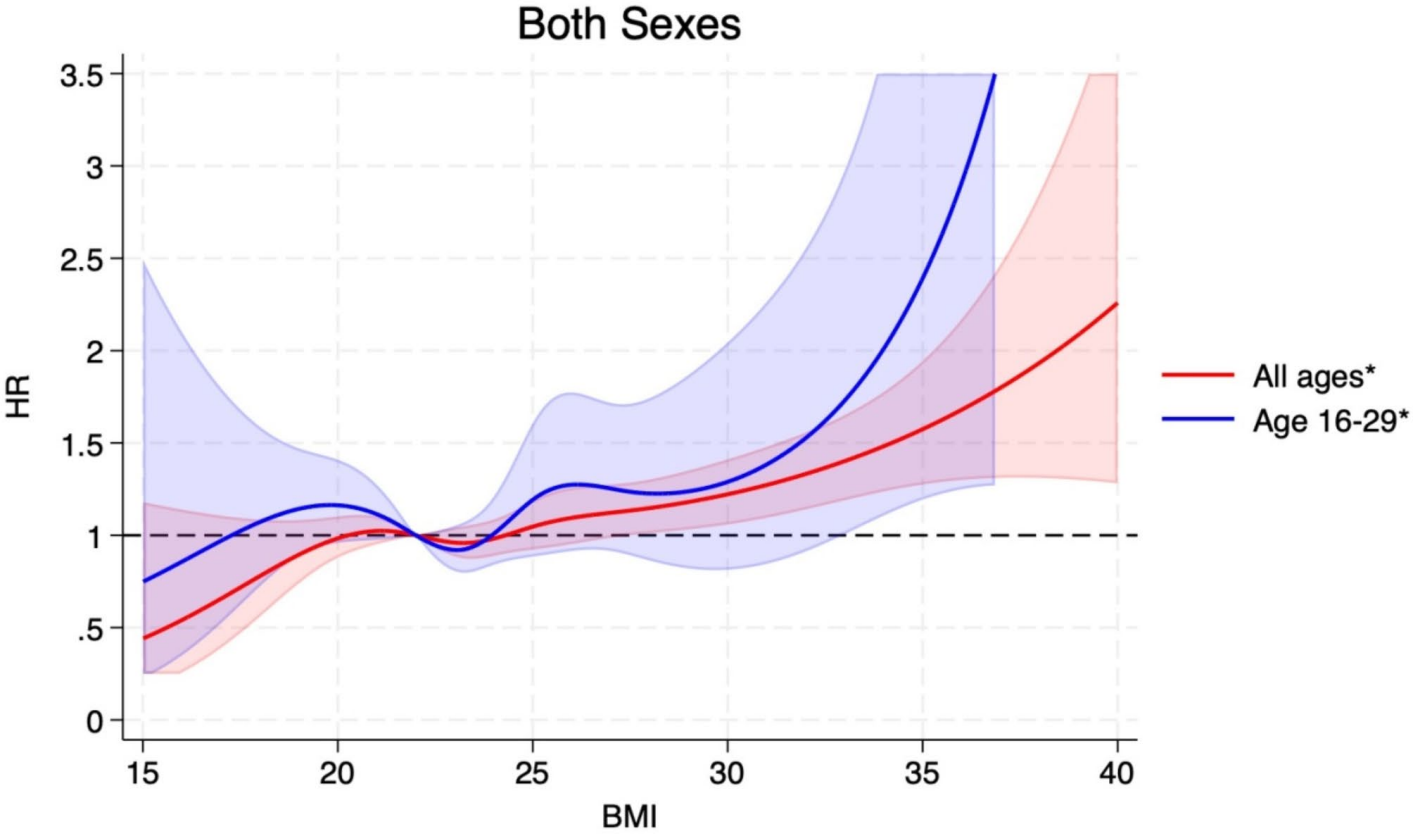
Restricted cubic splines for BMI and connective and soft tissue cancer in both sexes based on data from the Norwegian Tuberculosis Screening Program

**Fig. 2:**
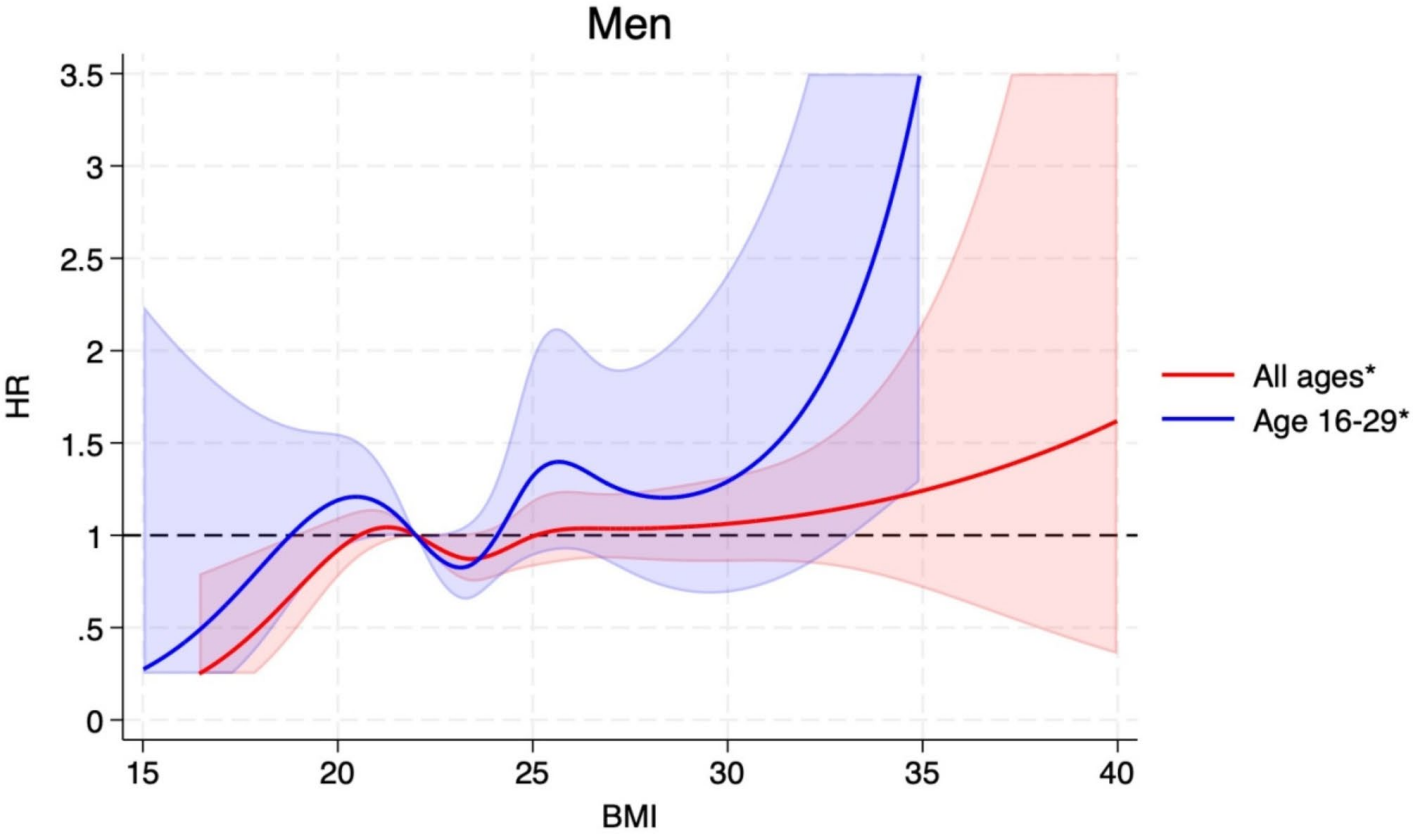
Restricted cubic splines for BMI and connective and soft tissue cancer in men based on data from the Norwegian Tuberculosis Screening Program

**Fig. 3:**
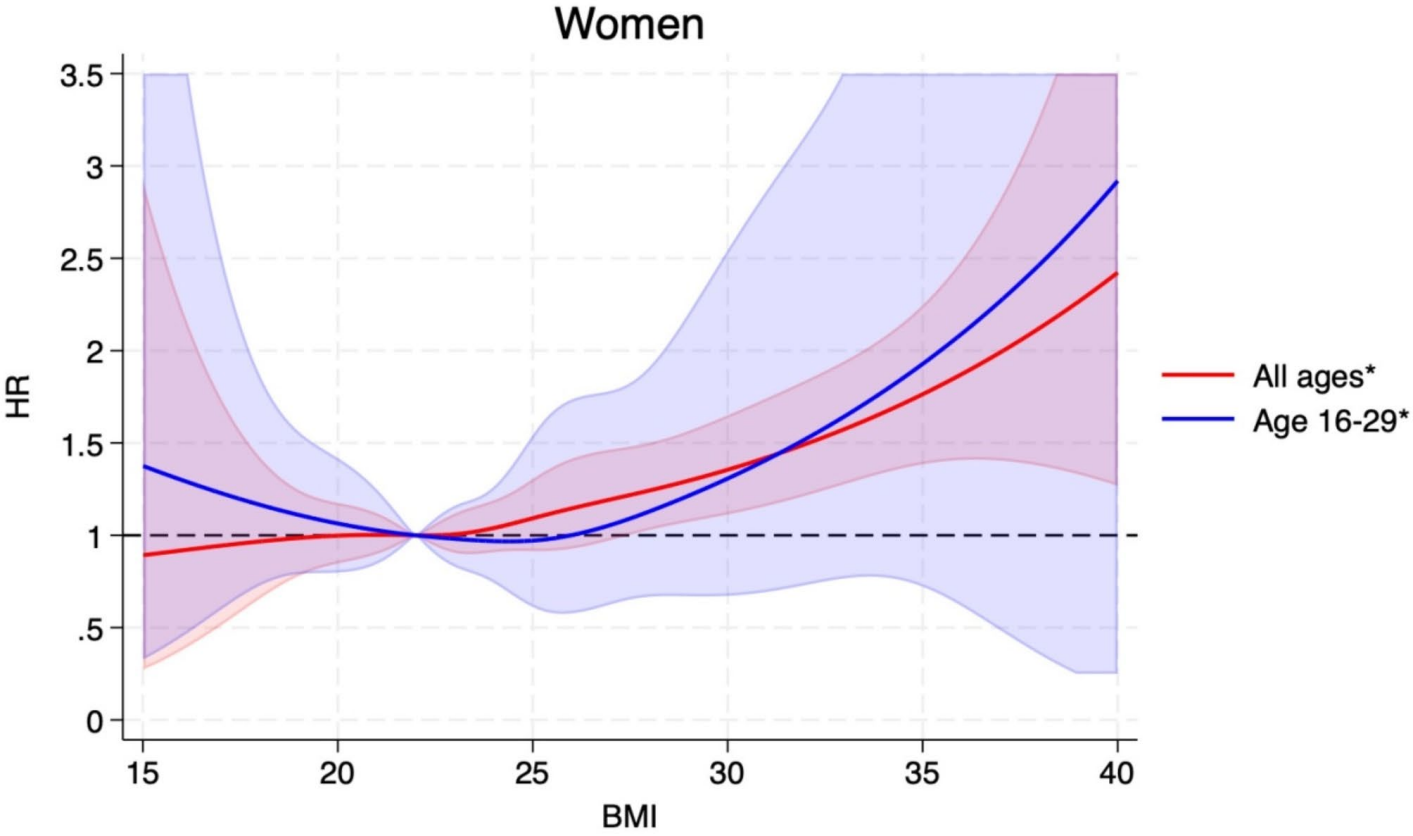
Restricted cubic splines for BMI and connective and soft tissue cancer in women based on data from the Norwegian Tuberculosis Screening Program

The HRs (95% CIs) for BMI categories of 15-<18.5, 18.5-<25.0 (ref.), 25.0-<30.0, 30.0-<35.0, ≥ 35.0 were 0.77 (0.50–1.20), 1.00, 1.15 (1.03–1.27), 1.28 (1.06–1.54), and 1.69 (1.21–2.37, p_trend_<0.0001), respectively, and per 5 kg/m^2^ increment in BMI was 1.14 (1.07–1.22) (Table 2). The corresponding estimates among men and women are also shown in Table 2. The positive associations observed persisted when excluding the first 5 years of follow-up (Table 2). Analyses using restricted cubic splines showed similar associations and there was no evidence of nonlinearity overall (*p* = 0.73), for men (*p* = 0.84), or for women (*p* = 0.22) (Figs. 1, 2 and 3). The distribution of cases by histological subtypes of connective and soft tissue cancer is shown in the Supplement. We observed positive associations between BMI (HRs, 95% CIs per 5 kg/m^2^ increment) and risk of soft tissue sarcoma (1.27, 1.09–1.50), fibrosarcoma (1.30, 1.02–1.66), dermatofibroma (1.28, 1.06–1.54)), lipoleiomyoma (1.24, 1.03–1.50), while no clear associations were observed with fibrous histiocytoma (1.06, 0.86–1.30) and liposarcoma (1.05, 0.92–1.22) (Supplementary Table 1).

**Table 2:** Hazard ratios (HRs) and 95% confidence intervals (95% CIs) for the association between body mass index categories and the risk of connective and soft tissue cancer

	Body mass index							
	15-<18.5	18.5-<25.0	25.0-<30.0	30.0-<35.0	≥ 35.0	≥ 30.0	Per 5 kg/m^2^	p_trend_
**All participants**	36,114	988,908	545,538	124,409	28,723	153,132	1,723,692	
Person-years	1,430,142	35,565,964	1,543,443	3,023,094	661,649	3,684,743	56,112,292	
Cases	21	955	612	134	36	170	1758	
HR (95% CI)^1^	0.77 (0.50–1.20)	1.00	1.15 (1.03–1.27)	1.28 (1.06–1.54)	1.69 (1.21–2.37)	1.35 (1.14–1.59)	1.14 (1.07–1.22)	< 0.0001
**Men**	12,297	494,357	282,710	36,563	3154	39,717	829,081	
Person-years	463,116	16,730,109	7,722,030	826,317	63,085	889,404	25,804,658	
Cases	5	537	349	35	5	40	931	
HR (95% CI)^2^	0.49 (0.20–1.19)	1.00	1.12 (0.97–1.29)	1.01 (0.72–1.43)	2.05 (0.85–4.94)	1.08 (0.78–1.49)	1.10 (0.99–1.23)	< 0.0001
**Women**	23,817	494,551	262,828	87,846	25,569	113,415	894,611	
Person-years	967,026	18,835,854	7,709,413	2,196,776	598,564	2,795,340	30,307,633	
Cases	16	418	263	99	31	130	827	
HR (95% CI)^2^	0.94 (0.57–1.56)	1.00	1.18 (1.01–1.39)	1.45 (1.16–1.82)	1.73 (1.19–2.50)	1.51 (1.23–1.85)	1.17 (1.08–1.27)	< 0.0001
**Exclusion of first 5 years of follow-up**								
**All participants**	35,660	973,194	534,356	121,569	28,019	149,588	1,692,798	
Person-years	1,419,262	35,218,181	15,258,324	2,988,097	653,615	3,641,713	55,537,480	
Cases	21	878	558	117	32	149	1606	
HR (95% CI)1	0.84 (0.54–1.29)	1.00	1.14 (1.02–1.27)	1.21 (0.99–1.47)	1.64 (1.14–2.34)	1.28 (1.07–1.53)	1.12 (1.04–1.20)	< 0.0001
**Men**	12,103	486,154	276,879	35,690	3077	38,767	813,903	
Person-years	459,783	16,580,417	7,640,381	817,593	62,483	880,076	25,560,656	
Cases	5	495	311	26	5	31	842	
HR (95% CI)2	0.54 (0.22–1.31)	1.00	1.08 (0.94–1.25)	0.82 (0.55–1.22)	2.25 (0.93–5.44)	0.91 (0.63–1.32)	1.05 (0.93–1.18)	0.02
**Women**	23,557	487,040	257,477	85,879	24,942	110,821	878,895	
Person-years	959,479	18,637,764	7,617,943	2,170,505	591,131	2,761,636	29,976,823	
Cases	16	383	247	91	27	118	764	
HR (95% CI)2	1.01 (0.61–1.68)	1.00	1.22 (1.03–1.44)	1.47 (1.16–1.86)	1.67 (1.12–2.47)	1.51 (1.22–1.87)	1.16 (1.07–1.27)	< 0.0001

^1^ Adjusted for age, sex

^2^ Adjusted for age

Obesity grade 2 in early adulthood was positively associated with connective and soft tissue cancer risk, with a HR 3.27 (1.05–10.21, p_trend_=0.09) when compared to normal BMI, however, the association was not statistically significant on a continuous scale with a HR of 1.11 (0.93–1.31) per 5 kg/m^2^ (Table 3). No association was observed between higher BMI and early-onset connective and soft tissue cancer (Table 4).

**Table 3:** Hazard ratios (HRs) and 95% confidence intervals (95% CIs) for the association between body mass index in early adulthood (age 16–29 years) and the risk of connective and soft tissue cancer

	Body mass index							
	15-<18.5	18.5-<25.0	25.0-<30.0	30.0-<35.0	≥ 35.0	≥ 30.0	Per 5 kg/m^2^	p_trend_
**All participants**	24,023	360,084	59,128	6681	1204	7885	451,120	
Person-years	1,107,445	16,706,096	2,702,766	299,102	52,977	352,078	20,868,387	
Cases^3^	18	294	61	-	-	11	384	
HR (95% CI)^1^	1.04 (0.65–1.67)	1.00	1.19 (0.90–1.56)	1.47 (0.73–2.97)	3.27 (1.05–10.21)	1.73 (0.95–3.16)	1.11 (0.93–1.31)	0.09
**Men**	8399	173,817	31,219	2536	257	2793	216,228	
Person-years	385,392	7,982,682	1,399,063	109,177	10,652	119,830	9,886,967	
Cases^3^	5	155	40	-	-	5	205	
HR (95% CI)^2^	0.78 (0.32–1.92)	1.00	1.33 (0.93–1.89)	1.33 (0.42–4.17)	9.77 (2.42–39.45)	2.03 (0.83–4.95)	1.19 (0.93–1.53)	0.04
**Women**	15,614	186,291	27,896	4144	947	5091	234,892	
Person-years	722,052	8,723,414	1,303,703	189,924	42,324	232,249	10,981,420	
Cases^3^	13	139	21	-	-	6	179	
HR (95% CI)^2^	1.17 (0.67–2.08)	1.00	0.97 (0.62–1.54)	1.57 (0.64–3.83)	1.40 (0.20–9.99)	1.54 (0.68–3.48)	1.03 (0.81–1.31)	0.88

^1^ Adjusted for age, sex

^2^ Adjusted for age

^3^ Counts have been suppressed for cells when *n* < 5 and some neighbouring cells with small numbers

**Table 4:** Hazard ratios (HRs) and 95% confidence intervals (95% CIs) for the association between body mass index and the risk of early-onset connective and soft tissue cancer

	Body mass index							
	15-<18.5	18.5-<25.0	25.0-<30.0	30.0-<35.0	≥ 35.0	≥ 30.0	Per 5 kg/m^2^	p_trend_
**All participants**	30,188	719,415	262,365	41,532	8760	50,292	1,062,260	
Person-years	778,335	13,764,202	3,346,724	449,362	88,938	538,300	18,427,562	
Cases^3^	6	166	44	-	-	7	223	
HR (95% CI)^1^	0.74 (0.32–1.67)	1.00	0.95 (0.67–1.34)	0.99 (0.44–2.26)	0.88 (0.12–6.35)	0.97 (0.45–2.10)	0.94 (0.75–1.17)	0.01
**Men**	9661	341,193	143,073	14,153	1167	15,320	509,247	
Person-years	271,933	6,546,056	1,816,270	162,017	14,181	176,198	8,810,458	
Cases^3^	-	87	32	-	0	-	123	
HR (95% CI)^2^	0.33 (0.05–2.43)	1.00	1.09 (0.72–1.67)	1.13 (0.36–3.60)	-	1.04 (0.33–3.32)	0.95 (0.68–1.32)	0.002
**Women**	20,527	378,222	119,292	27,379	7593	34,972	553,013	
Person-years	506,401	7,218,146	1,530,453	287,345	74,756	362,102	9,617,104	
Cases^3^	-	79	12	-	-	-	100	
HR (95% CI)^2^	0.93 (0.38–2.30)	1.00	0.69 (0.38–1.29)	0.91 (0.29–2.92)	1.16 (0.16–8.41)	0.96 (0.35–2.67)	0.93 (0.69–1.27)	0.56

^1^ Adjusted for age, sex

^2^ Adjusted for age

^3^ Counts have been suppressed for cells when *n* < 5 and some additional cells with small numbers

## Discussion

In this large Norwegian cohort study, we found a positive dose-response relationship between BMI and connective and soft tissue cancer risk. There was a 15%, 28% and 69% increase in risk among participants with overweight, obesity grade 1 and obesity grade 2, respectively, compared to individuals of normal weight. Moreover, there was a 14% increase in risk per 5 kg/m^2^ increment in BMI overall, and 17% and 10% increases in risk per 5 kg/m^2^ in women and men, respectively. The observed association persisted in sensitivity analyses excluding the first 5 years follow-up, suggesting reverse causation is not likely to explain the findings. A positive association was also observed for obesity in young adulthood. No association was observed between BMI and risk of early-onset connective and soft tissue cancer. When specific histological subtypes were investigated we observed positive associations with soft tissue sarcoma, fibrosarcoma, dermatofibroma, and lipoleiomyoma, but no clear associations with fibrous histiocytoma and liposarcoma.

To our knowledge this is the third large prospective cohort study to investigate the association between BMI and connective and soft tissue cancer incidence, but it is the first to investigate specific histologic subtypes. The findings are consistent observations made in a Swedish cohort which reported a 64% increased risk of connective tissue cancer among subjects with obesity vs. normal weight [16], and a pooled analysis of 15 Swedish cohorts reporting a 34% increased risk among subjects with obesity vs. those with normal weight [17]. The findings are also consistent with large registry-based retrospective cohort studies of hospital diagnoses of obesity from the US [13], Sweden [14], and Denmark [15], and with two case-control studies [18, 19] showing positive associations with risk of connective and soft tissue cancer.

Although adiposity has been related to a wide range of metabolic, immunologic and hormonal imbalances [31], it is unclear which mechanism(s) that may explain or contribute to the observed association between higher BMI and risk of connective and soft tissue cancer risk. A mice study showed that obesity-related immune dysfunction can increase the development of sarcomas when exposed to chemical carcinogens [32]. It has been speculated that accumulation of chemicals such as herbicides and pesticides in adipose tissue could contribute to increased risk of connective and soft tissue cancers [18]. Although adiposity is strongly associated with insulin resistance and type 2 diabetes [33], studies have so far not indicated any association between diabetes and connective tissue cancer risk [34–38], suggesting this may be a less likely explanation.

Obvious limitations of the current study is the absence of information on other factors that could confound the observed associations, such as smoking, physical activity, various lifestyle factors, or exposure to toxins and chemical carcinogens. Future studies incorporating more detailed information on these factors could provide further clarity. In addition, BMI is an imperfect measure of adiposity and does not distinguish between muscle and fat mass, however, BMI is still a strong predictor of adiposity-related diseases in population-based studies [39] as it is highly correlated with fat mass in most people. BMI was assessed at one time point only and we were therefore not able to take into account any changes in BMI over time. Other studies in Norway have documented increases in BMI over time at the population level [40], thus it is possible that weight gain could account for some of the increased risk observed. Lastly, we also had a smaller number of cancer cases in the analyses of BMI in early adulthood and for early-onset cancers, thus further studies are needed to clarify these findings. Notable strengths of the study include its large sample size, measured weight and height data by health professionals, comprehensive linkage to a complete and nationwide cancer registry, and an extensive follow-up period with minimal loss to follow-up. The current study covered approximately half the Norwegian population at the time of recruitment, thus it is likely that the current findings are broadly generalisable to the population at that time.

In summary, we found that overweight, and grade 1 and grade 2 obesity when compared to normal weight were associated with increased risk of connective and soft tissue cancer by 15%, 28% and 69%, respectively, and the associations were most pronounced for the histological subtypes soft tissue sarcoma, fibrosarcoma, dermatofibroma, and lipoleiomyoma. Additionally, a positive association was suggested with obesity in early adulthood, but BMI was not associated with early-onset disease. Large cohort studies with more detailed information on confounding factors and BMI trajectories over time are needed for further verification before firm conclusions can be made.

## Electronic supplementary material

Below is the link to the electronic supplementary material.

Supplementary Material 1

Supplementary Material 2

## Data Availability

Availability of data and materials: The dataset used for this study is not publicly available due to the conditions set out in the General Data Protection Regulation (GDPR) and Norwegian law. Data sharing provides legal basis and conditions set out in applicable law. Data from the Norwegian health registries can be applied for using www.helsedata.no.
