# Supplementary material for: Body mass index and risk of connective and soft tissue cancer: results from a large cohort of 1.7 million individuals in Norway
Source: BMC Cancer. 2025 Feb 11;25:243. doi: 10.1186/s12885-025-13637-8 (PMC11817836; doi:10.1186/s12885-025-13637-8)
Supplement: Supplementary file 2 — Supplementary Material 2 [file 12885_2025_13637_MOESM2_ESM.docx]

# Supplementary information for:

Aune D, Nordsletten M, Myklebust TÅ, Robsahm TE, Skålhegg BS, Mala T, Yaqub S, Saeed U. Body mass index and risk of connective and soft tissue cancer: results from a large cohort of 1.7 million individuals in Norway.

# Supplementary information on morphological codes

# Connective and soft tissue cancer

Diagnostic criteria: ICD10: C49 - 1758 cases

## Histological subtypes:

**Unsure benign/malignant: 65 cases**

**Carcinoma: 3 cases**

**Sarcoma*: 1690 cases**

***ICD-03 morphology number: Cases**

| 8800 | Soft tissue sarcom | 260 |
| --- | --- | --- |
| 8801 | Spindle cell carcinoma | 29 |
| 8810 | Fibrosarcoma | 110 |
| 8830 | Fibrous Histocytoma | 179 |
| 8832 | Dermatofibroma | 187 |
| 8890 | Leiomyoma | 190 |
| 8891 | Epithelioid leiomyoma | 1 |
| 8936 | GIST | 2 |
| 8990 | Mesenchyoma | 10 |
| 9120 | Hemangiosarcoma | 29 |
| 9130 | Angioendothelioma | 4 |
| 9133 | Epithelioid hemangioendothelioma | 3 |
| 9140 | Kaposi sarcoma | 52 |
| 9473 | Primitive neuroectodermal tumor | 1 |
| 9580 | Granular cell tumor | 6 |
| 8802 | Giant cell sarcoma | 12 |
| 8803 | Small cell carcinoma | 2 |
| 8804 | Epithelioid sarcoma | 2 |
| 8811 | Fibromyxoma | 46 |
| 8813 | Fascial fibroma | 2 |
| 8815 | Fibrous tumor | 5 |
| 8821 | Aggressive fibromatosis | 1 |
| 8840 | Myxoma | 8 |
| 8850 | Liposarcoma | 233 |
| 8851 | Liposarcoma | 91 |
| 8852 | Fibromyxolipoma | 27 |
| 8853 | Round cell liposarcoma | 5 |
| 8854 | Pleomorphic liposarcoma | 21 |
| 8855 | Mixed liposarcoma | 4 |
| 8858 | Dedifferntiated liposarcoma | 15 |
| 8900 | Rhabdomyoma | 37 |
| 8901 | Pleomorphic rhabdomyosarcoma | 9 |
| 8910 | Embryonal rhabdomyosarcoma | 1 |
| 8920 | Alveolar rhabdomyosarcoma | 1 |
| 8951 | Mesodermal mixed tumor | 1 |
| 8963 | Malignant rhabdoid tumor | 2 |
| 9040 | Synovioma | 50 |
| 9041 | Spindle cell synovial sarcoma | 1 |
| 9043 | Biphasic synovial sarcoma | 7 |
| 9044 | Clear cell sarcoma | 2 |
| 9150 | Hemangiopericytoma | 17 |
| 9180 | Osteosarcoma | 8 |
| 9220 | Chondoma | 9 |
| 9231 | Myxoid chondrosarcoma | 7 |
| 9250 | Osteoclastoma | 1 |

Supplementary Table 1. Hazard ratios (95% confidence intervals) for the association between body mass index and histological subtypes of connective and soft tissue cancer

| Histological subtype (morphological codes - ICD-O3) | Subset | | Body mass index categories | | | | | |  |
| --- | --- | --- | --- | --- | --- | --- | --- | --- | --- |
|  |  | | 15-<18.5 | 18.5-<25.0 | 25.0-<30.0 | 30.0-<35.0 | ≥35.0 | ≥30.0 | Per 5 kg/m^2^ |
| Soft tissue sarcoma (8800) | All | Cases^1^ | 0 | 123 | 108 | - | - | 29 | 260 |
|  |  | HR (95 CI) | - | 1.00 | 1.47 (1.13-1.92) | 1.92 (1.25-2.94) | 0.72 (0.17-2.94) | 1.73 (1.14-2.63) | 1.27 (1.09-1.50) |
|  | Men | Cases^1^ | 0 | 73 | 64 | - | - | 9 | 146 |
|  |  | HR (95 CI) | - | 1.00 | 1.46 (1.03-2.06) | 1.65 (0.79-3.44) | 2.93 (0.41-21.18) | 1.73 (0.86-3.49) | 1.29 (0.98-1.69) |
|  | Women | Cases^1^ | 0 | 50 | 44 | - | - | 20 | 114 |
|  |  | HR (95 CI) | - | 1.00 | 1.50 (0.99-2.27) | 2.08 (1.21-3.58) | 0.41 (0.06-3.02) | 1.73 (1.02-2.96) | 1.27 (1.04-1.56) |
| Fibrosarcoma (8810) | All | Cases^1^ | 0 | 56 | 45 | - | - | 9 | 110 |
|  |  | HR (95 CI) | - | 1.00 | 1.42 (0.95-2.13) | 1.10 (0.49-2.43) | 1.42 (0.34-5.92) | 1.15 (0.56-2.37) | 1.30 (1.02-1.66) |
|  | Men | Cases^1^ | 0 | 27 | - | - | 0 | - | 54 |
|  |  | HR (95 CI) | - | 1.00 | 1.54 (0.88-2.69) | 1.10 (0.26-4.69) | - | 1.02 (0.24-4.35) | 1.43 (0.93-2.20) |
|  | Women | Cases^1^ | 0 | 29 | - | - | - | - | 56 |
|  |  | HR (95 CI) | - | 1.00 | 1.28 (0.71-2.30) | 1.04 (0.39-2.72) | 1.50 (0.35-6.35) | 1.14 (0.49-2.65) | 1.24 (0.92-1.67) |
| Fibrous histiocytoma (8830) | All | Cases^1^ | - | 101 | 61 | - | - | 15 | 179 |
|  |  | HR (95 CI) | 0.80 (0.20-3.27) | 1.00 | 0.99 (0.72-1.37) | 0.79 (0.41-1.54) | 1.98 (0.80-4.92) | 0.99 (0.57-1.73) | 1.06 (0.86-1.30) |
|  | Men | Cases^1^ | 0 | 56 | 32 | - | 0 | - | 91 |
|  |  | HR (95 CI) | - | 1.00 | 0.92 (0.59-1.43) | 0.78 (0.42-2.49) | - | 0.72 (0.22-2.33) | 1.07 (0.75-1.52) |
|  | Women | Cases^1^ | - | 45 | 29 | - | - | - | 88 |
|  |  | HR (95 CI) | 1.25 (0.30-5.21) | 1.00 | 1.08 (0.67-1.75) | 0.84 (0.37-1.88) | 2.37 (0.93-6.05) | 1.14 (0.60-2.20) | 1.06 (0.82-1.38) |
| Dermatofibroma (8832) | All | Cases^1^ | - | 90 | 69 | 18 | 7 | 25 | 187 |
|  |  | HR (95 CI) | 1.46 (0.46-4.65) | 1.00 | 1.23 (0.90-1.70) | 1.64 (0.98-2.75) | 3.15 (1.44-6.91) | 1.88 (1.20-2.98) | 1.28 (1.06-1.54) |
|  | Men | Cases^1^ | - | 56 | 38 | - | 0 | - | 100 |
|  |  | HR (95 CI) | 1.43 (0.19-10.41) | 1.00 | 0.98 (0.65-1.48) | 1.13 (0.45-2.82) | - | 1.05 (0.42-2.63) | 1.00 (0.71-1.40) |
|  | Women | Cases^1^ | - | 34 | 31 | - | - | - | 87 |
|  |  | HR (95 CI) | 1.48 (0.35-6.23) | 1.00 | 1.76 (1.06-2.91) | 2.44 (1.26-2.72) | 4.88 (2.13-11.20) | 2.96 (1.66-5.26) | 1.48 (1.19-1.85) |
| Lipoleiomyoma (8890) | All | Cases^1^ | - | 103 | 63 | - | - | 21 | 190 |
|  |  | HR (95 CI) | 0.97 (0.30-3.08) | 1.00 | 1.10 (0.80-1.53) | 1.26 (0.72-2.20) | 2.41 (1.04-5.58) | 1.46 (0.89-2.37) | 1.24 (1.03-1.50) |
|  | Men | Cases^1^ | 0 | 56 | 31 | - | 0 | - | 88 |
|  |  | HR (95 CI) | - | 1.00 | 0.97 (0.62-1.53) | 0.29 (0.04-2.09) | - | 0.27 (0.04-1.95) | 0.97 (0.67-1.40) |
|  | Women | Cases^1^ | - | 47 | 32 | - | - | - | 102 |
|  |  | HR (95 CI) | 1.57 (0.48-5.10) | 1.00 | 1.26 (0.80--2.02) | 1.81 (0.98-3.35) | 2.98 (1.26-7.07) | 2.05 (1.19-3.54) | 1.36 (1.10-1.69) |
| Liposarcoma (8850, 8851, 8852, 8853, 8854, 8855, 8858) | All | Cases^1^ | 8 | 230 | 127 | 25 | 6 | 31 | 396 |
|  |  | HR (95 CI) | 1.14 (0.56-2.32) | 1.00 | 1.05 (0.84-1.32) | 1.08 (0.71-1.66) | 1.31 (0.58-2.96) | 1.12 (0.76-1.65) | 1.05 (0.92-1.22) |
|  | Men | Cases^1^ | - | 132 | 77 | - | - | 6 | 217 |
|  |  | HR (95 CI) | 0.74 (0.18-3.02) | 1.00 | 1.05 (0.78-1.41) | 0.62 (0.25-1.52) | 1.74 (0.24-12.50) | 0.69 (0.31-1.59) | 0.98 (0.78-1.24) |
|  | Women | Cases^1^ | - | 98 | 50 | - | - | 25 | 179 |
|  |  | HR (95 CI) | 1.37 (0.60-3.14) | 1.00 | 1.04 (0.73-1.49) | 1.39 (0.84-2.28) | 1.32 (0.53-2.82) | 1.37 (0.88-2.17) | 1.11 (0.93-1.33) |

^1^ Counts have been suppressed for cells when n<5 and for some additional cells.
